# Online lectures in undergraduate medical education: how can we do better?

**Published:** 2019-03-13

**Authors:** Brandon Tang, Alon Coret, Henry Barron, Aatif Qureshi, Marcus Law

**Affiliations:** 1 Faculty of Medicine, University of Toronto, Ontario, Canada; 2 Department of Family and Community Medicine, Faculty of Medicine, University of Toronto, Ontario, Canada

Medical education – much as the rest of the education world – has undergone significant change in the digital age. Whether through online textbooks and modules, computer-based exams, or video lectures, medical students today are expected to learn from a variety of technology-enabled modalities. Moreover, with the advent of these new modalities, many medical schools have shifted toward a “flipped classroom” model for learning.^1^^,^^2^ This approach calls on students to learn and prepare independently – often from self-guided, digitalized tools – before attending live classroom sessions. This cultural shift is calling on medical educators to rethink and revamp their pedagogical toolbox.

As medical students who are “coming of age” during this transitional time, we have had first-hand experience with the good and the bad of medical education, whether in-classroom or online. We have witnessed the ways in which “traditional” lectures can captivate an audience or induce narcolepsy, and conversely, the ways in which an online module can be an enjoyable exercise or a dreary chore. We would argue, however, that in many cases it is not the lecture content or the presenter’s efficacy that are at fault for poor learning outcomes, but rather the *inattentive design* of the presentation material.

There is, of course, a science to teaching: pedagogy. And in the medical education world, we are fortunate to have brilliant professors and researchers who – beyond their medical and scientific training – have taken it upon themselves to earn advanced degrees in education. What is perhaps lesser known is the pedagogy of *online* education, such as effective multimedia design. In his book, *Multimedia Learning*, educational psychologist Richard Mayer outlines key principles to guide the development of online materials (Table 1).^3^ Mayer’s work describes an evidence-based approach to improving learning outcomes from digitalized multimedia presentations. And, most importantly, there is evidence that these principles improve the *retention* and *transfer* of knowledge in core curricular medical school lectures.^4^

**Table 1:** Summary of Mayer’s multimedia design principles^5^

Principle	Definition^a^
*Coherence*	Exclude extraneous words, pictures and sounds
*Pre-Training*	Ensure students possess prior knowledge about names and characteristics of the main concepts
*Spatial Contiguity*	Present corresponding words and pictures in close proximity to one another
*Temporal Contiguity*	Present corresponding words and pictures simultaneously rather than successively
*Signalling*	Highlight important words
*Redundancy*	Pair animation and narration together without on-screen text
*Voice*	Use non-accented human spoken voice for narration over a machine simulated or foreign-accented human voice
*Personalization*	Employ conversational style, instead of formal, to present words
*Segmenting*	Offer narrated animation in learner-paced segments rather than a continuous unit
*Modality*	Pair animation and narration together instead of pairing animation and on-screen text

^a^Definitions reproduced from AAMC-IIME’s Effective Use of Educational Technology in Medical Education.^6^

While many of these principles may seem like common sense, it is surprisingly easy to find examples of where they are not applied, not applied well, or not applied with explicit intent. This is where our research comes in.

Our goal was to better understand how online education – and in particular, online lectures – has been utilized in medical schools internationally. To that end, we completed a scoping review on the topic. We included English-language articles about *undergraduate* medical education, with a focus on lectures that are *didactic* and whose primary purpose is to *teach* or *review* content. We specifically excluded other forms of online learning, namely non-lecture modalities such as case-based learning or interactive online modules.^7^

Our results ranged from confirmatory to startling. As we suspected, online lectures are employed broadly in medical schools around the world and incorporated into both basic sciences and clinical skills teaching. Their perception by students is generally positive. Alarmingly, of the 45 studies we included, a worrisome majority did not comment on any specific development strategy (44%) or simply rehashed previous recordings of live lectures (22%). Only three studies (7%) mentioned the intentional consideration of multimedia design principles as part of their development process.^7^

Our work confirms that medical education is adapting to the digital age, but raises concerns over the lack of a rational or evidence-based process through which this transition is occurring. It is not enough, we would argue, to simply digitalize lectures or flip classrooms; rather, this should be an evidence- and principle-based transformation. We need to make *best* practice the common practice.

The world of online learning is still relatively nascent, and many medical schools are in the process of adapting their classrooms to these cultural and technological reforms. It is our hope that medical educators and curriculum developers come to recognize the inherent potential of these new learning modalities and apply evidence-based design principles to the creation of online learning materials. As students and aspiring educators, we are excited to see what changes the coming century will bring to the training of tomorrow’s doctors.

## References

[1] Williams DE The future of medical education: flipping the classroom and education technology. The Ochsner Journal. 2016;16(1):14–5. 27026786 PMC4795492

[2] Schwartzstein RM, Roberts DH Saying goodbye to lectures in medical school—paradigm shift or passing fad?. New England Journal of Medicine. 2017;377(7):605-7. 28813217 10.1056/NEJMp1706474

[3] Mayer RE. Multimedia Learning. Cambridge University Press, 2001.

[4] Issa N, Schuller M, Santacaterina S et al Applying multimedia design principles enhances learning in medical education. Medical education. 2011;45(8):818-26. 21752078 10.1111/j.1365-2923.2011.03988.x

[5] University of Hartford Faculty Center for Learning Development. 12 Principles of Multimedia Learning; n.d [Internet]. Available at: http://hartford.edu/academics/faculty/fcld/data/documentation/technology/presentation/powerpoint/12_principles_multimedia.pdf [Accessed April 10, 2018].

[6] Candler C Effective use of educational technology in medical education. In Colloquium on educational technology: recommendations and guidelines for medical educators. Washington, DC: AAMC Institute for Improving Medical Education, 2007.

[7] Tang B, Coret A, Qureshi A, Barron H, Ayala AP, Law M Online lectures in undergraduate medical education: Scoping review. JMIR Med Educ. 2018;4(1):e11 29636322 10.2196/mededu.9091 PMC5915670

